# Urinary Sodium Excretion Enhances the Effect of Alcohol on Blood Pressure

**DOI:** 10.3390/healthcare10071296

**Published:** 2022-07-13

**Authors:** Xiyun Jiang, Mila D. Anasanti, Fotios Drenos, Alexandra I. Blakemore, Raha Pazoki

**Affiliations:** 1 Division of Biosciences, Department of Life Sciences, College of Health, Medicine and Life Sciences, Brunel University London, London UB8 3PH, UK; xiyunjiang@yahoo.com (X.J.); fotios.drenos@brunel.ac.uk (F.D.); alex.blakemore@brunel.ac.uk (A.I.B.); 2 Faculty of Information Technology, Nusa Mandiri University, Jakarta 10450, Indonesia; mila.mld@nusamandiri.ac.id; 3 Department of Metabolism, Digestion and Reproduction, Faculty of Medicine, Imperial College London, London W12 7TA, UK; 4 Department of Epidemiology and Biostatistics, School of Public Health, Imperial College London, London W12 7TA, UK

**Keywords:** genetics of alcohol, urinary sodium, cardiovascular traits

## Abstract

Alcohol consumption is linked to urinary sodium excretion and both of these traits are linked to hypertension and cardiovascular diseases (CVDs). The interplay between alcohol consumption and sodium on hypertension, and cardiovascular diseases (CVDs) is not well-described. Here, we used genetically predicted alcohol consumption and explored the relationships between alcohol consumption, urinary sodium, hypertension, and CVDs. Methods: We performed a comparative analysis among 295,189 participants from the prospective cohort of the UK Biobank (baseline data collected between 2006 and 2010). We created a genetic risk score (GRS) using 105 published genetic variants in Europeans that were associated with alcohol consumption. We explored the relationships between GRS, alcohol consumption, urinary sodium, blood pressure traits, and incident CVD. We used linear and logistic regression and Cox proportional hazards (PH) models and Mendelian randomization in our analysis. Results: The median follow-up time for composite CVD and stroke were 6.1 years and 7.1 years respectively. Our analyses showed that high alcohol consumption is linked to low urinary sodium excretion. Our results showed that high alcohol GRS was associated with high blood pressure and higher risk of stroke and supported an interaction effect between alcohol GRS and urinary sodium on stage 2 hypertension (P_interaction_ = 0.03) and CVD (P_interaction_ = 0.03), i.e., in the presence of high urinary sodium excretion, the effect of alcohol GRS on blood pressure may be enhanced. Conclusions: Our results show that urinary sodium excretion may offset the risk posed by genetic risk of alcohol consumption.

## 1. Introduction

Cardiovascular disease (CVD) is a global public health problem, killing 17 million people annually [1]. Alcohol consumption plays a role in the development of hypertension and CVD [2,3,4] and reducing alcohol consumption has been shown to lower blood pressure [5].

The sodium balance plays an important role in blood pressure regulation [6,7]. Urinary sodium excretion is associated with blood pressure [8]. We have previously shown that higher genetic risk of urinary sodium is associated with higher systolic blood pressure (SBP) and diastolic blood pressure (DBP) [9]. Alcohol consumption has been reported to decrease urinary sodium excretion [10]. A recent cross-sectional study [11] used data from older adults in northern China and found that the combination of alcohol consumption and sodium intake imposed a greater risk of hypertension. This implies that there might be a synergic effect between alcohol consumption and sodium on the risk of hypertension.

Advances in genetic data acquisition and analysis have improved our understanding of complex relationships and the biological mechanisms underpinning complex diseases. Recent genome-wide association studies (GWASs) among Europeans identified genetic variants in the form of single nucleotide polymorphisms (SNPs) associated with alcohol consumption [12,13] and urinary sodium [9].

To better understand the relationships between alcohol consumption, urinary sodium, hypertension, and CVDs, we constructed a genetic risk score (GRS) for alcohol consumption based on 105 SNPs associated with alcohol consumption in Europeans [12,13]. We explored the relationship between GRS and alcohol consumption and the effect of GRS on blood pressure, risk of hypertension, and CVDs in relation with urinary sodium excretion. We performed our study using individual-level data from 295,189 UK Biobank (UKB) participants.

## 2. Materials and Methods

### 2.1. Ethics Approval

Ethical approval was obtained centrally by the UKB from the UKB Research Ethics Committee and Human Tissue Authority. All participants whose data was used in this study gave informed consent. We additionally obtained ethics approval from Brunel University London to work on secondary data from the UKB (25527-A-Jun/2021-32860-1).

### 2.2. Study Population and Exclusion Criteria 

**Figure 1:**
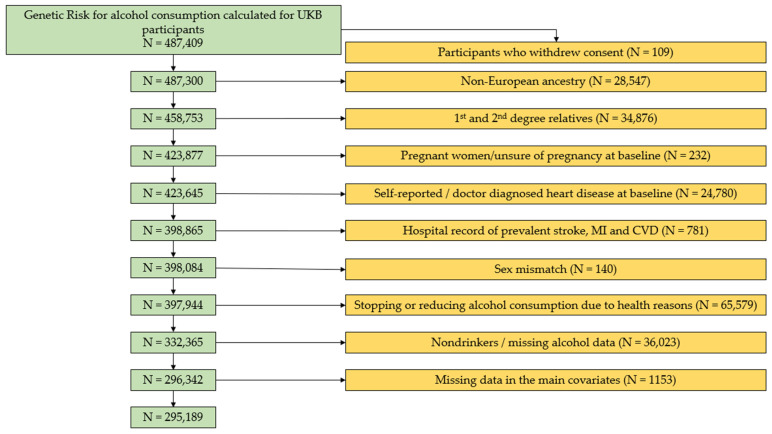
Exclusion flowchart. Exclusion criteria and the final UKB sample size used in the analysis. MI, myocardial infarction; CVD, cardiovascular diseases.

UKB is a large prospective cohort set up in 22 centers across the United Kingdom. It consists of over 500,000 participants aged 40 to 69 recruited between 2006 and 2010 [14]. Genetic data on 487,409 participants were available for analysis. We applied several exclusion criteria (Figure 1). We excluded participants who withdrew consent (*N* = 109), participants of non-European ancestry (*N* = 28,547), first- or second-degree relatives (*N* = 34,876), pregnant women or women unsure of pregnancy status at baseline (*N* = 232), prevalent CVD cases (*N* = 25,561), sex mismatch (*N* = 140), participants with health-related change in drinking habits (*N* = 65,579), non-alcoholic drinkers or participants with missing alcohol consumption data (*N* = 36,023), and participants with missing data regarding the main study variables (*N* = 1153). A total of 295,189 participants remained for analysis. Participants with health-related change in drinking habits included participants who self-reported to reduce/stop their drinking for one of the following reasons on a touch screen question: (1) illness or ill health, (2) doctor’s advice, or (3) health precaution.

### 2.3. Blood Pressure and Definition of Hypertension

Supplementary Table S1 lists the UKB data fields used in our analyses. Blood pressure was measured centrally by the UKB [15]. We have described details about the blood pressure phenotype previously [16]. In brief, for every participant, SBP and DBP was each measured twice at the UKB assessment center by an automated device (Omron HEM-7015IT digital BP monitor) or a manual device when automated readings could not be taken. Where multiple values for blood pressure existed, we used the mean of all available measurements. We adjusted the blood pressure for participants taking blood-pressure-lowering medication by adding 15 mmHg to their SBP and 10 mmHg to their DBP measurements according to Tobin et al. [17].

We defined hypertension as stage 1 or stage 2 according to the American Heart Association (AHA) guideline [18]. Stage 1 hypertension includes individuals with SBP between 130 and 139 mmHg or DBP between 80 and 89 mmHg [18]. Stage 2 hypertension includes individuals with SBP ≥ 140 mmHg and/or DBP ≥ 90 mmHg or using blood-pressure-lowering medications [18].

### 2.4. Cardiovascular Diseases 

We have described the definitions and methods for assessment of CVD events in detail previously [16]. In summary, CVD was defined as an episode of stroke, myocardial infarction, or coronary heart disease. 

Nonfatal and fatal records of CVD were extracted from Hospital Episode Statistics (HES). We used the International Classification of Diseases 9 and 10 codes provided in Supplementary Table S2 to extract CVD cases. We used the HES-recorded episode date as the date of CVD event or death. If the episode date was missing, we used the hospital admission date. For participants with multiple hospital admissions for the same condition, we used the first recorded date. 

Prevalent CVD cases were defined as a hospital record of CVD cases that occurred prior to the UKB baseline assessment date for each participant. Prevalent CVD cases were additionally identified from the UKB self-reported questionnaire at the baseline assessment for participants who self-reported previous diagnosis of CVDs (Supplementary Table S2). Incident cases were defined as newly diagnosed cases after the UKB baseline assessment date. The follow-up time for each participant was defined as the time from the UKB baseline assessment date for each participant until 31 March 2015. 

### 2.5. Assessment of Lifestyle Factors 

Urinary sodium excretion was measured from spot urine samples collected at the UKB baseline assessment center [19]. The concentration of sodium in the urine was measured by ion-selective electrode analysis using a Beckman Coulter AU5400. 

We calculated the alcohol intake in grams per day (g/day) for each UKB participant based on self-reported alcohol intake from the UKB touchscreen questionnaire at the baseline assessment. We used answers from questions on the alcohol intake frequency and average weekly intake of red wine, white wine, beer/cider, spirits, and fortified wine, respectively (Supplementary Table S1). Details of our alcohol intake (g/day) calculation were described previously [12]. In brief, for participants with complete responses to the alcohol questions listed above, we multiplied the quantity of the average weekly intake of each alcoholic beverage by its standard drink size and reference alcohol content. We then summed up the drink-specific intake based on the reported drinking frequency and converted alcohol consumption to gram per day.

As alcohol consumption was not normally distributed, we transformed alcohol consumption (g/day) on the natural logarithm scale. All subsequent mentions of alcohol consumption in this study refer to transformed alcohol consumption. 

Smoking status was recorded based on a UKB self-reported questionnaire [19]. Methods of assessment for sedentary lifestyle were mentioned in our previous publication [16]. We measured the sum of hours per day each participant spent sitting (watching TV, driving, and using a computer). Greater values indicated more hours spent sedentary.

To assess participants’ dietary intake, we calculated a Dietary Approaches to Stop Hypertension (DASH) score based on methods published elsewhere [16,20]. In brief, the DASH score was calculated using the UKB self-reported food questionnaire at baseline. We scored and ranked the participants based on the selected dietary components listed in Supplementary Table S1 and derived an overall DASH score for each participant. 

The Townsend deprivation score used UK national census data on car ownership, household overcrowding, owner-occupation, and unemployment for each UK region/area [21]. The UKB assigned each participant a score based on the residence postcode the participant provided at the baseline assessment.

### 2.6. Genotyping, Imputation, and Genetic Calculations in the UKB

Genotyping and imputation were conducted centrally by the UKB. Detailed methodologies were provided elsewhere [14,15]. In brief, participants’ blood samples were collected at the UKB assessment center and DNA was extracted and genotyped using the UKB Axiom Array. Genotype imputation used 3 reference panels, including Haplotype Reference Consortium, UK10K, and 1000 Genomes phase 3. The imputation was performed using the IMPUTE4 program. UKB calculated the genetic principal components and kinship coefficients centrally. These were used to account for population stratification and to identify related individuals [14].

### 2.7. GRS for Alcohol Consumption 

**Figure 2:**
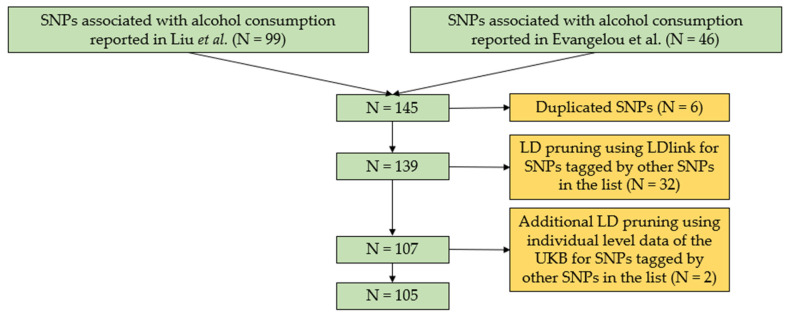
SNPs selection flowchart for the alcohol consumption genetic risk score. Flowchart detailing the SNPs selection process prior to being used in the calculation of the alcohol consumption genetic risk score for UKB participants. We obtained SNPs identified for alcohol consumption from Liu et al. [13] (99 SNPs) and Evangelou et al. [12] (46 SNPs). We first removed duplicates (*n* = 6). We then assessed LD (R^2^ > 0.1) among the SNPs and identified LD pairs using the LDmatrix function in LDlink [22]. Within each SNP pair, SNPs with a stronger association with alcohol consumption were moved forward (32 SNPs removed). The remaining SNPs underwent extra LD pruning using individual-level data of the UKB (see methods; 2 SNPs removed). Eventually, 105 SNPs were selected to calculate the alcohol genetic risk score. SNP, single nucleotide polymorphism; LD, linkage disequilibrium; UKB, UK Biobank.

We calculated a GRS for alcohol consumption based on 105 published SNPs (Supplementary Table S3) associated with alcohol consumption in Europeans [12,13]. The SNP selection process is illustrated in Figure 2. To summarize, we obtained SNPs (*N* = 145) identified from two large-scale GWAS meta-analyses on alcohol consumption [12,13]. After removing duplicates, we assessed the linkage disequilibrium (LD) among the remaining SNPs using LDlink [22] and PLINK version 1.9 (Shaun Purcell, Christopher Chang, Boston, MA, USA, URL:www.cog-genomics.org/plink/1.9/ accessed on 12 July 2022) [23]. We defined SNP pairs with R^2^ > 0.1 as correlated SNPs. Within each SNP pair, we removed the SNP with the weaker association with alcohol consumption as indicated by a larger *p* value. As a result of these exclusions, 105 SNPs (Supplementary Table S3) remained for the alcohol GRS calculation. 

To calculate the GRS for each UKB participant, we sought the effect estimates for the 105 alcohol SNPs from the alcohol GWAS meta-analysis study by Liu et al. [13]. To avoid sample overlap with the UKB data, we specifically extracted the effect estimates from the summary statistics excluding UKB and 23andMe provided by Liu et al. [13]. We also checked that this summary statistics dataset used the same genome build assembly (GRCh37) as the UKB genotype data [14]. To calculate the GRS, we multiplied the effect estimates by the number of risk alleles each UKB participant carries on the alcohol SNPs. The products were then summed across all SNPs to produce an overall weighted GRS for each participant. We standardized the weighted GRS for further analysis.

### 2.8. Secondary Analyses

To better understand the etiology behind our findings, we performed a series of secondary analyses, including regression analyses, between urinary sodium and various alcoholic beverages and various outcomes such as diabetes, myocardial infarction, stroke, and CVD. We additionally performed Mendelian randomization (MR) analysis between urinary sodium and alcohol consumption. MR uses genetic variants (SNPs) that are robustly associated with an exposure of interest as instrumental variables to assess the causal effect of the exposure on an outcome [24]. In our analysis, the frequency of alcohol consumption was considered as the exposure and urinary sodium was considered as the outcome. The alcohol SNP selection process is illustrated in Figure 2. To further process these SNPs for MR analysis, we clumped these SNPs at a distance = 10,000 kb and R^2^ = 0.001 [25] to ensure the independence of SNPs according to the MR guidelines. This removed 27 additional SNPs. Consequently, to identify any weak alcohol instruments that could lead to weak instrument bias in the MR analysis [26], a parameter called F-statistics was used, where if <10 indicates a weak instrument [27,28]. We calculated F-statistics using a published formula [29]:

$$F=R^{2}\left(N-2\right)/\left(1-R^{2}\right)$$

where *R*^2^ is the variance in alcohol consumption explained by each SNP and was calculated using a published formula [30]; *N* is the size of the sample in which SNP–alcohol consumption association test statistics were calculated. After removal of the weak instruments, we were eventually left with 33 SNPs for the two-sample MR analysis. We obtained the corresponding test statistics for the association of these SNPs with urinary sodium within the UKB from Pazoki et al. [9]. We performed SNP harmonization across the alcohol and urinary sodium datasets and checked the strand orientation prior to analysis. To derive the MR estimates, we used the inverse variance weighted (IVW) method, which calculates the MR causal estimate with the highest precision [31]. However, these methods assume no horizontal pleiotropy. Horizontal pleiotropy occurs when the SNPs affect urinary sodium through traits other than alcohol consumption and these traits are not in the causal pathway from alcohol consumption to urinary sodium. When horizontal pleiotropy is left unbalanced, it can bias the MR causal estimate [32]. To detect the presence of unbalanced horizontal pleiotropy and correct the MR estimate for any bias due to this, we performed additional sensitivity tests such as the MR-Egger [32], weighted median [33], and the weighted mode tests [34]. An MR-Egger intercept *p* value < 0.05 suggests overall unbalanced horizontal pleiotropy [32]. To claim significance on MR analyses, we used a *p* value threshold of 0.05.

### 2.9. Statistical Analysis 

We assessed the variance in alcohol consumption explained by GRS using the adjusted R^2^ estimate from a linear regression model regressing alcohol consumption on GRS. To assess the predictability of the GRS at different alcohol consumption levels, we compared the percentage variation in alcohol consumption explained by the GRS across alcohol consumption quintiles comprising 5 equal groups.

We investigated the association of the GRS with SBP, DBP, Stage 1 hypertension, Stage 2 hypertension, and incident CVDs. We used linear regression for SBP and DBP, logistic regression for Stage 1 and Stage 2 hypertension, and performed survival analysis using Cox proportional hazards (PH) regression that takes the follow-up time into account for incident CVD traits. In our survival analyses, individuals who were lost to follow-up, died of diseases not under study, or did not develop diseases of interest at the end of the follow-up were censored. We assessed the PH assumption for every Cox model using statistical tests that used Schoenfeld residuals [35] against the follow-up time. When the *p* value for the PH assumption global test was <0.05, i.e., the overall PH assumption was violated, we examined which specific covariate violated the PH assumption in the model. For time-varying continuous covariate(s), we added interaction terms, with the follow-up time split into groups. The interaction term was modeled on a multiplicative scale in a linear regression model for blood pressure, logistic model for hypertension, and Cox model for CVDs.

We tested two statistical models in all analyses. We adjusted model 1 for age, age^2^, and sex. In model 2, we additionally adjusted for major known cardiovascular and genetic confounders, including smoking status, DASH diet, Townsend deprivation score, sedentary lifestyle, and genetic principal components. We adjusted the analyses for the interaction between alcohol GRS and urinary sodium, where the interaction term was statistically significant.

### 2.10. Power Calculation 

We calculated the statistical power for the associations of alcohol GRS with hypertension and CVDs using Quanto (version 1.2.4, Los Angeles, CA, USA) [36,37]. Using a two-sided significance threshold of 0.05 and a range of a number of cases and effect estimates from 0.9–1.3, we obtained an estimation of the statistical power for our analyses.

### 2.11. Software and Packages 

We calculated alcohol GRS using PLINK version 1.9 [23]. We conducted all statistical analyses in R studio (R version: 3.5.1, Vienna, Austria). We used the ‘survival’ package for survival analysis and ‘TwoSampleMR’ package for the causal inference analysis.

## 3. Results

We included 295,189 UKB participants in the current analysis after applying the exclusion criteria (Figure 1). At baseline (Table 1), participants had a mean age of 56.3 years at recruitment. Approximately 54.5% of the sample were female (*N* = 161,020). Stage 1 hypertension was present among 23.1% of the sample (*N* = 68,284; 23.5% male vs. 22.8% female) while stage 2 hypertension was present in 52% of the sample (*N* = 153,474; 59.4% male vs. 45.8% female). The median follow-up time for composite CVD and stroke was 6.1 and 7.1 years, respectively. During the follow-up time, 8688 participants (2.9%) developed CVD and 1857 (0.6%) developed stroke.

**Figure 3:**
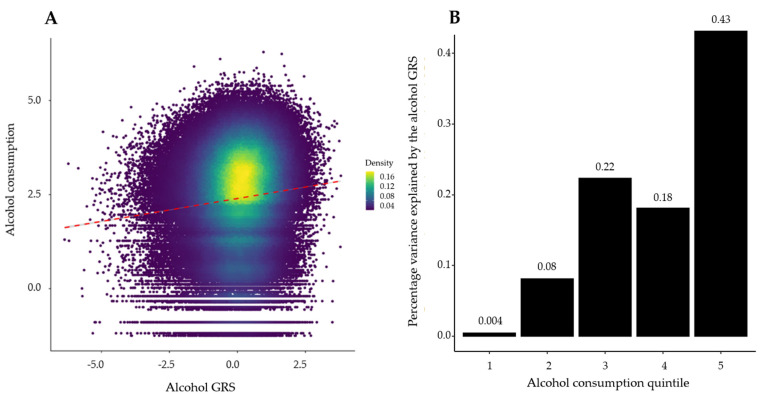
Relationship between the alcohol genetic risk score (GRS) and daily alcohol consumption. (**A**) Scatterplot illustrates the relationship between alcohol consumption (on a natural logarithm scale) and alcohol GRS. The red dotted line shows the gradient of the fitted regression line. (**B**) Percentage variation in alcohol consumption explained by the alcohol GRS (adjusted R^2^ estimate) within alcohol consumption quintiles. Quintile 1: 0 to 3.65 g/day; Quintile 2: 3.6–10.7 g/day; Quintile 3: 10.7–17.9 g/day; Quintile 4: 17.9–31.3 g/day; Quintile 5: 31.3–964.5 g/day alcohol consumption.

One standard deviation (SD) addition of alcohol GRS corresponded to a 0.12-fold increase in alcohol consumption (natural logarithm transformed equivalent to an increase in alcohol consumption by 1.13 g/day; Figure 3A). GRS explained ~1% of the variation in alcohol consumption (Figure 3). 

Within the whole UKB sample (*N* = 295,189), we observed an association of the GRS with SBP (β = 0.24 mmHg; 95% CI = 0.17, 0.31; *p* = 2.73 × 10^−11^), DBP (β = 0.13 mmHg; 95% CI = 0.09, 0.17; *p* = 8.56 × 10^−11^), stage 2 hypertension (odds ratio = 1.02; 95% CI = 1.01, 1.03; *p* = 1.92 × 10^−8^), and stroke (hazard ratio= 1.06; 95% CI = 1.01, 1.11; *p* = 0.01). Similar results were observed in the sex-specific analysis (Supplementary Table S4).

**Figure 4:**
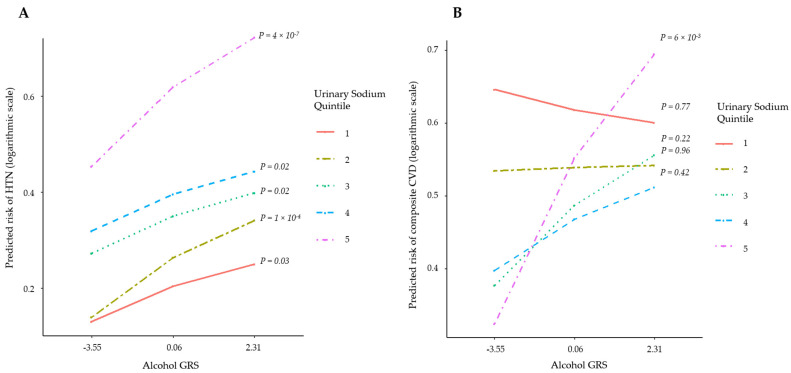
Effect of the alcohol genetic risk score (GRS) on (**A**) hypertension and (**B**) cardiovascular disease across urinary sodium excretion quintiles. X axis shows the median values of GRS in GRS quintile 1, 3, and 5, respectively: −3.549, 0.058, and 2.312.

We observed an interaction between alcohol GRS and urinary sodium on stage 2 hypertension (P_interaction_ = 0.03) and CVD (P_interaction_ =0.03) (Table 2). Among participants with high urinary sodium excretion, alcohol GRS showed a greater effect on hypertension and CVD (Figure 4).

**Figure 5:**
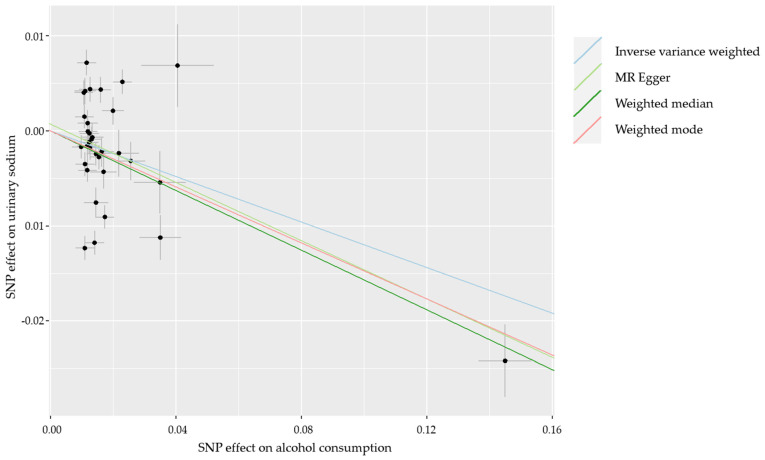
Two-sample MR for the relationship between alcohol consumption and urinary sodium. Lines represent regression line for various MR tests used. Black dots with cross represent each SNP. Effect of SNPs on alcohol consumption and urinary sodium are in the logarithmic scale. SNP, single nucleotide polymorphism; MR, Mendelian randomization.

In our secondary analyses to better understand the identified relationships, we observed that urinary sodium was linked to more frequent hypertension, diabetes, and CVDs (Supplementary Table S5). We additionally found that alcohol consumption is associated with lower urinary sodium (Supplementary Table S5). Additionally, using MR to infer causality between alcohol consumption and urinary sodium, we confirmed that an increase in alcohol consumption decreased urinary sodium levels (random-effect IVW: β = −0.120; 95% CI = −0.216, −0.023; *p* = 0.015; Figure 5). 

## 4. Discussion

In this large-scale longitudinal study, we used data from 295,189 alcohol drinkers and provided insight into the interplay between genetic factors, alcohol consumption, and urinary sodium on blood pressure and cardiovascular outcomes. We found that the genetic factors underpinning alcohol consumption are linked to higher blood pressure and stroke. Alcohol consumption lowers urinary sodium and the effect of genetic factors on stage 2 hypertension and the risk of CVD is greater among participants with high urinary sodium excretion.

Observational studies have demonstrated an association of alcohol consumption (drinks per day) with blood pressure [38] and CVD [39]. Similarly genetic epidemiological studies [40,41] focusing on genetic variants (rs671/rs1229984) within the aldehyde/alcohol dehydrogenase (ALDH2/ADH1B) gene demonstrated that individuals carrying the risk allele for alcohol consumption had a higher risk of hypertension [40,41] and CVD [40] compared to non-carriers. Our results additionally capture variation in the genetic underpinning of alcohol consumption and show that 105 variants identified thus far for alcohol consumption collectively (in the form of a GRS) are associated with higher blood pressure and risk of stroke. Moreover, our results suggest that a gradient increase in blood pressure and CVD due to genetic risk may be altered by changes in sodium intake. We showed that higher GRS remains associated with higher blood pressure at all levels of urinary sodium excretion. However, at lower vs. higher level of urinary sodium, GRS makes a smaller difference in blood pressure.

High alcohol consumption, high alcohol GRS [42], and a higher sodium concentration are all known to be linked to high blood pressure [8]. In a previously published study [9] using the LD score regression method [43], we showed a genetic correlation exists between urinary sodium excretion and the frequency of alcohol consumption. Our results in the current study move a step forward and show a causal relationship, as confirmed by MR analysis, between alcohol consumption and urinary sodium excretion. A decrease in urinary sodium upon administration of ethanol was observed since decades ago [44]. For example, rats who were sedated using ethanol had a lower urinary sodium and higher plasma concentration of sodium compared to the control group. A study on human participants in the early 1990s showed that after the ingestion of alcohol, urinary sodium is lower whilst the sodium concentration in red blood cells is higher compared with the control group [10]. This suggests that ethanol has an impact on sodium retention, and it likely occurs through the kidneys as sodium retention in plasma occurs at the same time as a decrease in urinary sodium excretion. In our causal inference analysis using MR, we found similar findings and showed that alcohol consumption decreases urinary sodium. We identified an interaction between urinary sodium and alcohol GRS on hypertension in such a way that alcohol GRS has a higher effect on blood pressure among participants with high urinary sodium excretion. It is likely that participants with higher urinary sodium excretion have a higher capacity for sodium retention as the kidneys have access to a larger pool of urinary sodium when exposed to alcohol and, thus, these participants show a larger increase in blood pressure. 

We should note that there are challenges involved in the estimation of sodium intake from urinary sodium excretion. Although reducing sodium intake is important in reducing blood pressure, monitoring sodium intake in patients is difficult. Most methods of estimating sodium intake rely on self-reported data and are at risk of recall bias and these methods require a nutritionist to interpret them. The collection of 24 h urinary sodium has been used for the estimation of sodium intake, but it is difficult for patients to collect, especially in an accurate way. Equations have been created and used to estimate sodium intake from sodium in spot urine [45]. However, these methods have been recently deemed as biased by the European Salt Action Network [46]. Therefore, although spot urine is generally regarded as a reliable proxy to reflect salt intake, we have been cautious in this study to generalize results using the spot urinary sodium to sodium intake. 

The large sample size of the UKB in addition to its rich phenotyping provides optimal statistical power to investigate the relationship between genetic factors and complex outcomes. The use of individual-level data allowed us to investigate the effect of potential confounders and perform subgroup analyses. Additionally, our GRS for alcohol consumption was created based on a total of 105 SNPs identified from the two largest-scale GWAS meta-analysis studies for alcohol consumption thus far [12,13]. This is the largest number of SNPs identified for alcohol consumption so far and allowed us to capture more variation in alcohol consumption compared to previous studies that used a limited number of SNPs in their analyses. Yet, these SNPs (although the largest currently available) capture less than 1% of the total variation in alcohol consumption [13]. Future large-scale GWASs would be helpful to increase the number of SNPs identified for alcohol consumption. One of our limitations is that UKB is a recently established study and thus has a relatively short follow-up time to study outcomes and this might have affected the statistical power for investigation of the survival data. It should be considered that alcohol consumption is assessed using a self-reported questionnaire in the UKB and thus bias due to underreporting or over-reporting may be present. It has been shown that self-reported alcohol data can be more reliable if collected within a shorter time span prior to the completion of questionnaires [47]. The UKB has self-reported data available on the average monthly intake and average weekly intake of alcohol. To reduce recall bias related to self-reported alcohol data, we used the weekly intake of alcohol instead of monthly intake. Another limitation of our study was the fact that we used spot urine samples as this is the only available source to quantify sodium excretion within the UKB while the gold standard for sodium intake estimation is a 24 h collection or timed overnight collections. Sample handling and storage of the urine samples was carried out centrally by the UKB. A spot urine sample was obtained at the time of the clinic visit. These were scheduled throughout the day so samples would have been obtained over the same period and timing was, therefore, not standardized. A most recent study [48] has demonstrated that if drawn from a random and representative sample of the population, the spot urinary sodium concentration accurately reflects sodium excretion drawn from 24 h urine collection. 

## 5. Conclusions

Our study shows that the genetic underpinning of alcohol consumption in the form of a GRS is linked to high blood pressure and a higher risk of stroke and that the effect of GRS is greater among participants with a higher amount of urinary sodium excretion. Our current findings suggest the role of a reduction in sodium intake and alcohol consumption to decrease blood pressure. Our study informs health policies in terms of alcohol consumption and its interactions. Patients with high urinary sodium excretion should be cautioned on the potential enhanced impact of alcohol consumption on hypertension.

## Figures and Tables

**Table 1:** Baseline characteristics and incident events of cardiovascular diseases for participants of the UKB stratified by sex.

	Overall (*N* = 295,189)	Males (*N* = 134,169)	Females (*N* = 161,020)
Age at recruitment, mean (SD), years	56.3 (8)	56.4 (8.1)	56.2 (7.9)
Males, *N* (%)	134,169 (45.5)	NA	NA
Smoking, *N* (%)			
Current	30,388 (10.3)	16,341 (12.2)	14,047 (8.7)
Past	148,209 (50.2)	70,575 (52.6)	77,634 (48.2)
Never	116,592 (39.5)	47,253 (35.2)	69,339 (43.1)
Healthy diet score (DASH), mean (SD)	2.7 (1)	2.4 (1)	2.9 (1)
Sedentary lifestyle, median [IQR), hours/day	4 (3, 6)	5 (3, 6)	4 (3, 5)
SBP *, median (IQR), mmHg	138.5 (125.5, 153.5)	142 (130, 156)	135 (122, 150.5)
DBP *, mean (SD), mmHg	84.2 (11.2)	86.5 (11)	82.2 (11)
Stage 1 Hypertension ^†^, *N* (%)	68,284 (23.1)	31,492 (23.5)	36,792 (22.8)
Stage 2 Hypertension ^‡^, *N* (%)	153,474 (52)	79,680 (59.4)	73,794 (45.8)
Townsend Deprivation Index, median (IQR)	−2.4 (−3.8, −0.06)	−2.4 (−3.8; −0.02)	−2.4 (−3.8; −0.1)
Urinary sodium, median (IQR) ^§^	68 (42.7, 102.8)	81.9 (53.4, 117.5)	57.6 (36.5, 88.3)
Composite cardiovascular disease, *N* (%)	8688 (2.9)	5808 (4.3)	2880 (1.8)
Stroke, *N* (%)	1857 (0.6)	1048 (0.8)	809 (0.5)

* SBP and DBP adjusted for blood-pressure-lowering medication. ^†^ Stage 1 hypertension defined as SBP 130–139 mmHg or DBP 80–89 mmHg. ^‡^ Stage 2 hypertension defined as SBP ≥ 140 mmHg and/or DBP ≥ 90 mmHg or using blood-pressure-lowering medication. ^§^ Urinary sodium had missing values and so the baseline statistics for urinary sodium were calculated based on an overall sample size of *N* = 286,806, men *N* = 130,894 and women *N* = 155,912. SBP, systolic blood pressure; DBP, diastolic blood pressure; DASH, Dietary approaches to stop hypertension; NA, not available; *N*, sample size IQR, interquartile range.

**Table 2:** Overview of the association of alcohol GRS with blood pressure, hypertension, and incident CVDs.

	Regression Model	*N* Non-Cases/ *N* Cases	Effect Estimates *	95% CI	*p* Value for GRS ^†^	*p* Value for Interaction Term	PH ^‡^
SBP	Linear	286,806	0.25	0.18, 0.32	3.12 × 10^−12^	0.052	NR
DBP	Linear	286,806	0.14	0.10, 0.19	3.50 × 10^−12^	0.33	NR
Stage 1 Hypertension	Logistic	71,322/66,395	1.01	1.00, 1.02	0.10	0.32	NR
Stage 2 Hypertension	Logistic	137,717/149,089	1.02	1.02, 1.03	9.78 × 10^−9^	0.03	NR
Stroke	Cox PH	285,012/1794	1.06	1.01, 1.11	0.02	0.72	0.23
Composite CVD	Cox PH	278,418/8388	1.01	0.99, 1.03	0.37	0.03	0.45

GRS and urinary sodium values were centered around the mean. * β value is given for linear models, odds ratios are provided for logistic models, and hazard ratios are presented for Cox models. ^†^ Models were fitted with model 2 adjustments and an interaction between alcohol GRS and urinary sodium. ^‡^ Overall *p* value for the PH assumption test from the Cox proportional hazard model derived from Schoenfeld residuals. *N*, sample size; GRS, genetic risk score; CI, confidence interval; PH, proportional hazard; SBP, systolic blood pressure; NR, not relevant; DBP, diastolic blood pressure; CVDs, cardiovascular diseases.

## Data Availability

Not applicable.

## Supplementary Materials

The following supporting information can be downloaded at: https://www.mdpi.com/article/10.3390/healthcare10071296/s1, Table S1: UK Biobank data fields for blood pressure, physical and lifestyle variables used in the analysis, Table S2: Codes used to generate cardiovascular disease phenotypes in the analysis, Table S3: Genetic variants used to construct the genetic risk score for alcohol consumption, Table S4: Sex-specific effect of alcohol genetic risk on blood pressure and cardiovascular diseases, Table S5: Overview of association of urinary sodium with alcohol consumption and various outcomes.
