# Supplementary material for: Urinary Sodium Excretion Enhances the Effect of Alcohol on Blood Pressure
Source: Healthcare (Basel). 2022 Jul 13;10(7):1296. doi: 10.3390/healthcare10071296 (PMC9319523; doi:10.3390/healthcare10071296)
Supplement: Supplementary file 1 [file healthcare-10-01296-s001.zip › healthcare-1758177-supplementary.pdf]

Supplemental Materials

Urinary sodium excretion enhances the effect of alcohol on blood pressure

Xiyun Jiang, MSc; Mila D. Anasanti, PhD; Fotios Drenos, PhD; Alexandra I. Blakemore, PhD; Raha Pazoki, MD, PhD

Table S1. UK Biobank data fields for blood pressure, physical and lifestyle variables used in the analysis.

**Variable name**	**UK Biobank data field(s)**
Alcohol intake (g/day)	1558, 1588,1578,1608,1568,1598
Systolic blood pressure*	4080,93
Diastolic blood pressure*	4079,94
Urinary sodium	30530
Smoking status	20116
Sedentary lifestyle	1070, 1090, 1080
Dietary approaches to stop hypertension (DASH)	1309, 1319, 1299, 1289, 1329, 1339, 1408, 1349, 1369, 1379, 1389
Townsend deprivation score	189

*****We manually adjusted systolic and diastolic blood pressure for participants taking blood pressure medication by adding 15 mmHg to their systolic blood pressure measurement and 10 mmHg to their diastolic blood pressure measurement.[16]

Table S2. Codes used to generate cardiovascular disease phenotypes in the analysis.

Phenotype	Code
Fatal and nonfatal CVD (HES data)	**ICD10:** I20, I21, I22, I23, I24, I25, I61, I60, I63, I64, I50, I42
	**ICD 9:** 428, 410, 411, 412, 413, 414, 4297, 431, 430, 434, 436,425
	**OPCS:** K40, K401, K402, K403, K404, K408, K409, K41, K411, K412, K413, K414, K418, K419, K42, K421, K422, K423, K424, K428, K429, K43, K431, K432, K433, K434, K438, K439, K44, K441, K448, K449, K45, K451, K452, K453, K454, K455, K458, K459, K46, K461, K462, K463, K464, K468, K469, K442, K456, K465, K471, K49, K491, K492, K493, K494, K498, K499, K50, K501, K504, K508, K509, K75, K751, K752, K753, K754, K758, K759
Prevalent CVD excluded	**UKB data field 20002:** 1081, 1082, 1583, 1086, 1491, 1066, 1067, 1087, 1492, 1591, 1074, 1075, 1076, 1079
	**UKB data field 20004:**1071, 1102, 1103, 1104, 1105, 1107, 1108, 1109, 1110, 1515, 1095, 1070
	**UKB data field 6150:** 1,2,3

CVD, Cardiovascular disease; HES, Hospital Episode Statistics; ICD, International Classification of Diseases; OPCS, Classification of Interventions and Procedures; UKB, UK Biobank. Table adapted from Pazoki et al.[15]

Table S3. Genetic variants used to construct the genetic risk score for alcohol consumption.

SNP	Chromosome	Position	Effect allele	Effect estimates	SE
rs10753661	1	165119792	A	-0.0071	0.0032
rs12031875	1	71585097	G	0.0035	0.0039
rs227179	1	210216731	G	0.0051	0.0030
rs2310752	1	66392405	A	-0.0045	0.0030
rs28680958	1	173848808	A	-0.0133	0.0037
rs58107686	1	33837334	A	-0.0126	0.0035
rs7517344	1	50711961	A	0.0086	0.0040
rs780569	1	4569436	A	-0.0018	0.0033
rs823114	1	205719532	A	0.0098	0.0030
rs10496076	2	57942987	C	0.0079	0.0031
rs11692435	2	98275354	A	0.0067	0.0050
rs1260326	2	27730940	C	0.0140	0.0031
rs13024996	2	144225215	A	-0.0088	0.0031
rs13032049	2	63581507	G	0.0160	0.0033
rs13383034	2	45155276	T	0.0115	0.0031
rs13390019	2	97797680	C	-0.0060	0.0046
rs2178197	2	27860551	G	-0.0022	0.0030
rs56337305	2	225475560	C	-0.0106	0.0031
rs72859280	2	147956293	T	0.0349	0.0083
rs77165542	2	430975	T	-0.0134	0.0089
rs785293	2	53023304	G	0.0049	0.0030
rs828867	2	74334462	A	0.0068	0.0030
rs13066454	3	93994255	T	-0.0045	0.0031
rs13094887	3	70968431	T	-0.0069	0.0033
rs2011092	3	141124607	C	-0.0016	0.0031
rs60654199	3	141267295	A	-0.0218	0.0064
rs62250685	3	85457240	G	-0.0124	0.0031
rs6787172	3	158187811	G	-0.0117	0.0030
rs9838144	3	131576287	C	-0.0065	0.0036
rs10004020	4	152968372	A	0.0029	0.0033
rs10028756	4	100254520	A	-0.0068	0.0046
rs11940694	4	39414993	G	0.0229	0.0030
rs1229984	4	100239319	C	0.1450	0.0085
rs12499107	4	99678691	G	0.0028	0.0043
rs12646808	4	3249828	C	-0.0067	0.0032
rs13107325	4	103188709	T	-0.0350	0.0066
rs16854020	4	42117559	A	0.0136	0.0046
rs2165670	4	100286085	A	0.0256	0.0046
rs36052336	4	100273594	G	-0.0163	0.0063
rs3748034	4	3446091	T	-0.0169	0.0042
rs4690727	4	143648579	G	0.0117	0.0033
rs4699791	4	101243023	A	0.0023	0.0050
rs7698119	4	171070910	A	-0.0082	0.0030
rs79139602	4	100444363	T	0.0076	0.0103
rs10078588	5	166816176	A	0.0086	0.0030

SNP	Chromosome	Position	Effect allele	Effect estimates	SE
rs12655091	5	144412335	A	-0.0108	0.0030
rs4916723	5	87854395	C	-0.0093	0.0030
rs55872084	5	155902003	T	0.0199	0.0036
rs10085696	7	69783020	G	-0.0145	0.0038
rs10249167	7	98980879	G	-0.0120	0.0046
rs34060476	7	73037956	G	0.0080	0.0045
rs35034355	7	103840115	A	-0.0092	0.0030
rs6951574	7	153489744	C	0.0087	0.0030
rs1217091	8	64527399	C	0.0111	0.0038
rs13250583	8	20949917	T	-0.0096	0.0036
rs2356369	8	64956882	C	0.0074	0.0030
rs28601761	8	126500031	G	0.0123	0.0030
rs55932213	9	108755622	G	0.0089	0.0034
rs74424378	9	109331094	G	-0.0053	0.0035
rs17665139	10	125093880	T	-0.0122	0.0042
rs61873510	10	102626510	T	-0.0063	0.0032
rs7074871	10	110507806	A	-0.0101	0.0034
rs12795042	11	133658168	C	-0.0131	0.0031
rs1713676	11	113660576	G	-0.0070	0.0030
rs2071305	11	47370957	C	-0.0092	0.0032
rs485425	11	121544984	G	0.0062	0.0030
rs4938230	11	116075001	A	0.0120	0.0042
rs7121986	11	113355444	C	0.0110	0.0031
rs748919	11	133783232	C	-0.0061	0.0039
rs7950166	11	8642218	T	-0.0094	0.0031
rs988748	11	27724745	G	0.0089	0.0036
rs10506274	12	81601464	T	-0.0076	0.0030
rs10876188	12	51895882	T	-0.0080	0.0030
rs12312693	12	57511734	C	0.0039	0.0030
rs4842786	12	92170791	A	-0.0145	0.0030
rs57281063	12	54660427	A	0.0089	0.0030
rs7958704	12	51984349	C	0.0011	0.0030
rs500321	13	27124360	T	-0.0110	0.0034
rs112635299	14	94838142	T	-0.0405	0.0116
rs11625650	14	104610138	A	-0.0101	0.0036
rs2277499	14	57271127	T	-0.0089	0.0031
rs71414193	14	58685301	A	-0.0115	0.0038
rs12907323	15	86796012	G	0.0079	0.0030
rs2472297	15	75027880	T	0.0088	0.0034
rs1104608	16	73912588	C	-0.0071	0.0030
rs113443718	16	29892184	A	-0.0083	0.0032
rs11648570	16	72356964	C	0.0151	0.0049
rs1421085	16	53800954	C	-0.0109	0.0030
rs17177078	16	24810681	T	-0.0187	0.0060
rs2764771	16	20013793	A	0.0085	0.0032
rs378421	16	28754684	A	-0.0154	0.0030
rs62044525	16	64872590	G	-0.0163	0.0039

SNP	Chromosome	Position	Effect allele	Effect estimates	SE
rs7185555	16	69131281	C	-0.0170	0.0042
rs10438820	17	78524597	T	0.0118	0.0033
rs1053651	17	37822311	C	0.0036	0.0033
rs1991556	17	44083402	A	-0.0089	0.0037
rs2854334	17	29715500	G	0.0119	0.0030
rs3803800	17	7462969	G	0.0101	0.0036
rs4548913	17	2209888	A	-0.0042	0.0031
rs4794015	17	47067826	A	0.0076	0.0030
rs4092465	18	55080437	G	-0.0125	0.0031
rs9320010	18	53053897	G	-0.0070	0.0031
rs281379	19	49214274	A	0.0173	0.0030
rs4815364	20	25035711	A	0.0073	0.0031
rs9607814	22	41946519	A	-0.0066	0.0040

SNP location, effect alleles, effect estimates, and SE were taken from the GWAS (Genome-wide association study) meta-analysis by Liu et al. for alcohol consumption (logarithm transformed alcoholic drinks per week) combining the GSCAN (GWAS & Sequencing Consortium of Alcohol and Nicotine use) cohorts but excluding the UK Biobank and 23andMe. SNP, single nucleotide polymorphism; SE, standard error.

Table S4. Sex-specific effect of alcohol genetic risk on blood pressure and cardiovascular diseases.

		N non-cases/ N cases	Effect estimate/ OR/HR *	95% CI	*P* value
**SBP, mmHg**					
**Model 1**	Men	134,169	0.38	0.28,0.47	8.98×10^{-14}
	Women	161,020	0.24	0.14,0.33	1.40×10^{-6}
**Model 2**	Men	134,169	0.30	0.20,0.40	4.50×10^{-9}
	Women	161,020	0.18	0.08,0.26	2.83×10^{-4}
**DBP, mmHg**					
**Model 1**	Men	134,169	0.19	0.13,0.25	4.38×10^{-10}
	Women	161,020	0.12	0.07,0.18	1.29×10^{-5}
**Model 2**	Men	134,169	0.16	0.10,0.22	1.04×10^{-7}
	Women	161,020	0.11	0.05,0.16	8.55×10^{-5}
**Hypertension Stage 1**					
**Model 1**	Men	22,997/31,492	1.01	1.00,1.03	0.14
	Women	50,434/36,792	1.01	1.00,1.02	0.13
**Model 2**	Men	22,997/31,492	1.01	0.99,1.03	0.39
	Women	50,434/36,792	1.01	0.99,1.02	0.24
**Hypertension Stage 2**					
**Model 1**	Men	54,489/79,680	1.04	1.03,1.05	5.37×10^{-11}
	Women	87,226/73,794	1.02	1.01,1.03	5.07×10^{-4}
**Model 2**	Men	54,489/79,680	1.03	1.02,1.05	1.35×10^{-8}
	Women	87,226/73,794	1.01	1.00,1.02	0.02
**Stroke**					
**Model 1**	Men	133,121/1,048	1.08	1.01,1.15	0.02
	Women	160,211/809	1.09	1.02,1.17	0.02
**Model 2**	Men	133,121/1,048	1.06	0.99,1.13	0.08
	Women	160,211/809	1.07	1.00, 1.15	0.06
**CVDs**					
**Model 1**	Men†	128,361/5,808	1.03	1.00,1.06	0.03
	Women	158,140/2,880	1.03	0.99,1.07	0.15
**Model 2**	Men	128,361/5,808	1.02	0.99,1.05	0.16
	Women	158,140/2,880	1.02	0.98,1.06	0.37

*Effect estimates were derived from multivariate linear regression (for systolic blood pressure and diastolic blood pressure), logistic regression (for hypertension), and multivariate Cox proportional hazards regression (for stroke and CVDs) models. Odds ratio represents the risk of hypertension for each standard deviation increase in the alcohol genetic risk score. †Model additionally fitted with an interaction between age and follow-up time. N, sample size; OR Odds Ratio; HR, Hazard Ratio; CI, confidence intervals; CVDs, Cardiovascular diseases; SBP, Systolic blood pressure; DBP, Diastolic blood pressure.

Table S5. Overview of association of urinary sodium with alcohol consumption and various outcomes.

	Effect Estimate	95% CI	P-value
**Stage 1 hypertension ***	0.09	0.08, 0.1	3.7×10^{-50}
**Stage 2 hypertension ***	0.16	0.15, 0.17	5.3×10^{-282}
**Diabetes, diagnosed by doctor ***	0.15	0.13, 0.17	9.9×10^{-42}
**MI ***	0.10	0.06, 0.13	2.4×10^{-07}
**Stroke ***	0.04	-0.01, 0.09	1.5×10^{-01}
**CVD ***	0.08	0.05, 0.1	3.8×10^{-11}
**Fatal CAD ***	0.12	0.03, 0.2	7.3×10^{-03}
**Average weekly red wine intake, glasses †**	-0.0138	-0.014, -0.013	0
**Average weekly champagne plus white wine intake, glasses †**	-0.0139	-0.014, -0.013	0
**Average weekly beer plus cider intake, pints †**	-0.0136	-0.014, -0.013	0
**Average weekly spirits intake, measures †**	-0.0143	-0.015, -0.014	0
**Average weekly fortified wine intake, glasses †**	-0.0139	-0.014, -0.013	0
**Total alcohol consumption (g/day)**	-0.0143	-0.015, -0.014	0

* Effect estimate is given for the effect of urinary sodium excretion on cardiovascular phenotypes from a logistic model adjusted for age, and sex. † Effect estimate is given for the effect of weekly consumption of alcoholic beverages (exposure) on urinary sodium (outcome). The linear model is adjusted for age and sex. CAD, coronary artery disease; CVD, cardiovascular diseases; MI, myocardial infarction
